# Three-Dimensional Segmentation and *in silico* Comparison of Equine Deep Digital Flexor Tendon Pathology in Horses Undergoing Repeated MRI Examination

**DOI:** 10.3389/fvets.2021.706046

**Published:** 2021-10-21

**Authors:** Kimberly D. Trolinger-Meadows, Adam H. Biedrzycki, Hongjia He, Natasha Werpy

**Affiliations:** ^1^Department of Large Animal Clinical Sciences, College of Veterinary Medicine, University of Florida, Gainesville, FL, United States; ^2^Equine Diagnostic Imaging, Inc., Archer, FL, United States

**Keywords:** 3D, segmentation, reconstruction, magnetic resonance imaging (MRI), horse, DDFT, tendon

## Abstract

The use of magnetic resonance imaging (MRI) has led to increased clinical and research applications using 3D segmentation and reconstructed volumetric data in musculoskeletal imaging. Lesions of the deep digital flexor tendon (DDFT) are a common pathology in horses undergoing MRI. Three-dimensional MRI reconstruction performed for volumetric tendon analysis in horses has not previously been documented. The aim of this proof-of-concept study was to evaluate the 3D segmentation of horses undergoing repeated MRI at several time points and to perform an analysis of the segmented DDFTs across time. MRI DICOM files were acquired from six horses undergoing repeated MRI examination of the foot for DDFT injury. Once segmented, volumetric tendon surface tessellation language (STL) files were created. Thickness and volumetric data were acquired for each tendon in addition to a tendon comparison across timepoints within each horse. Pearson correlation coefficients were calculated for comparison of MRI reports to computer analysis. There was a significant and positive correlation between MRI and medial record reports of clinical improvement or deterioration and computer analysis (*r* = 0.56, *p* = 0.01). The lower end range limit for tendon thickness varied between 0.16 and 1.74 mm. The upper end range limit for DDFT thickness varied between 4.6 and 23.6 mm. During tendon part comparison, changes in DDFT were reported between −3.0 and + 14.3 mm. Changes in DDFT size were non-uniform and demonstrated fluctuations throughout the tendon. The study was successful in establishing the volumetric appearance and thickness of the DDFT as it courses in the foot and tracking this over time. We encountered difficulties in accurate segmentation of the distal insertion of the DDFT as it blends with the distal phalanx. The data demonstrated that the DDFT can be segmented and volumetric studies based on size and shape can be performed using an *in silico* approach.

## Introduction

Lesions of the deep digital flexor tendon (DDFT) are a common pathology and are reported to occur in 82.6% of limbs in horses undergoing magnetic resonance imaging (MRI) evaluation (1). Lesions most commonly occur at the level of the collateral sesamoidean ligament or at the level of the navicular bone (1). MRI and Computed Tomography (CT) have been utilized to quantitatively assess the DDFT within the hoof capsule using two dimensional (2D) cross sectional area (2). Ultrasonographic evaluation for quantitative assessment of the deep digital flexor tendon proximal to the hoof capsule has also been described (3, 4). However, changes in cross sectional area depend on the type of DDFT pathology present and are not are not consistent. They can be contingent on the type of lesion present but also could depend on the modality used to determine the cross-sectional area (2).

Segmentation of medical images has become an indispensable process to perform quantitative analysis of images of biological structures (5). Computed tomography (CT) segmentation has been utilized for quantitative assessment in diagnosis, surgical planning, and post-operative assessment in orthopedic surgery (6). Volumetric CT scans have a short acquisition time and result in hundreds to thousands of image slices (6). However, soft tissue structures are challenging to segment and evaluate utilizing these CT based images (6). Soft tissue detail is more accurately evaluated with superior contrast resolution using ultrasound and magnetic resonance imaging (6, 7).

The cornerstone of musculoskeletal MRI is multiplanar 2D acquisition. Although the analysis of MRI provides a series of 2D images using a variety of imaging sequences, the focus has been on speed of image acquisition, to minimize anesthesia and imaging times, and using an MRI sequence which is optimal to highlight a lesion. As such, fewer image slices are made using an MRI compared to a CT examination. Since fewer slices are made with an MRI scan compared to a CT scan of the same region, each slice must either be thicker in the MRI scan or there is an increased distance between two slices. These criteria are important in determining the final make up of a 3D data point (voxel). The width and height of a voxel can be calculated by the following formula (8):

Voxel width (or height) in mm = Field of view (mm) / Matrix width (or height).

Where “matrix width/height” refer to the number of the number of pixels in the image along the horizontal (*x*-axis) and vertical (*y*-axis) axes respectively (9). The depth (*z*-axis) of a voxel is the distance between two slices, which can also be called as slice increment. When the length, width and depth are identical, the voxel is isotropic; when they are not, the voxel is anisotropic (9). Although the majority of equine MRI sequences are anisotropic and can be used for 3D analysis, isotropic voxels are better suited and preferred for 3D reconstruction.

Multiplanar 2D acquisition using sequences such as proton density (PD) or proton density fat saturated (PD FS) results in cuboidal shaped voxels that are typically five to nine times larger than the in-plane resolution, creating a predisposition to averaging of MR signal along the slice direction. This typically manifests at anatomical contrast margins and may obscure or eliminate potentially clinically relevant details. As innovations in imaging have led to the introduction of new technologies, 3D MRI sequences have been increasingly incorporated into human clinical musculoskeletal protocols. High-resolution isotropic 3D (HiRes-3D) MRI acquisitions have isotropic, or near isotropic voxels as well as a slice thickness equivalent to in-plane resolution. Therefore, one of the primary advantages of HiRes-3D sequences when compared to traditional multiplanar 2D fast spin-echo (FSE) techniques is a reduction in partial volume averaging artifacts (10).

The advent of HiRes-3D MRI acquisitions has led to increased clinical and research applications of 3D MRI in musculoskeletal imaging over the past decade in human medicine (11, 12). In human athletes, segmentation has been utilized to gather data that would be beneficial for injury prevention (13, 14). One study found that changes in volume of the anterior cruciate ligament are associated with injury (13). Another study identified a volumetric increase in size of the anterior cruciate ligament over the course of a competitive soccer season in female athletes (14).

The use of MRI for 3D reconstruction of anatomy has been previously performed for anatomical analysis of the metacarpophalangeal joint in horses (15). Additionally, the use of CT for 3D reconstruction of anatomy has been performed for anatomical description of the synovial structures in the equine distal limb (16). Image fusion of CT and MRI images has been described for development of a 3D musculoskeletal model of the equine forelimb (17). However, the use of MRI for 3D reconstruction for volumetric tendon analysis in horses has not previously been documented. The aim of this proof-of-concept study was to evaluate the 3D segmentation of horses undergoing repeated MRI at multiple time points and perform part analysis of the segmented tendons at the different time points. We hypothesized that thickness and volumetric measurement of the DDFT over time would correlate with changes in reported clinical lameness and pathological findings on MRI reports.

## Materials and Methods

Medical records of horses which were presented for high field (1.5 T) MRI examinations of the foot at the University of Florida and Equine Diagnostic Imaging, Inc. from 2015 to 2020 were reviewed. MRI studies were included if the MRI report findings included a lesion of the DDFT (reported by a board-certified veterinary radiologist) and if one or more repeat MRI examination were performed. Based on the history, it was noted if the left or right limb were clinically affected/abnormal at each time point and if there had been any clinical improvement since the previous examination. From the clinical comments and the MRI reports, changes in lameness and changes in MRI interpretation, focused on the DDFT only, were coded as follows: improvements were recorded as +1, no change as 0 and a worsening or progression as a −1. Comments regarding any effusion, changes to the navicular bone or collateral structures or any other changes between scans not related to the DDFT were excluded.

### MRI Study Acquisition and Evaluation

Initial MRI studies were performed using a 1.5-Tesla magnet (Vantage™, Toshiba, Tustin, CA). Follow-up MRI examination was performed with either the 1.5-Tesla magnet (Vantage™, Toshiba, Tustin, CA) or 0.27-Tesla magnet (sMRI, Hallmarq Advanced Veterinary Imaging, West Chicago, IL). MRI studies performed using the 1.5-Tesla magnet were performed under general anesthesia with the horse in lateral recumbency using a 160 mm coil (QD Knee Coil, Toshiba, Tustin, CA). Images extracted from these cases included the anisotropic Proton Density Weighted (PDW) and Proton Density Fat Saturation (PD FS) sequences. MRI studies performed using the 0.27-Tesla magnet were performed utilizing chemical restraint with the horse standing. Images extracted from these cases include both the anisotropic proton density weighted (PDW) and T1 weighted 3D (T1W 3D) isotropic sequences. MRI protocols varied between patients; however, all MRI studies included scan sequences obtained in sagittal, axial, and dorsal planes. All images were viewed using standard dedicated Digital imaging and communication in medicine files (DICOM) viewing software (Merge PACS™, IBM Watson Health, Chicago, IL or Keystone Omni, Asteris, Inc., Monument, CO). Information regarding image acquisition was obtained from the metadata of the DICOM files and included slice thickness, repetition time, echo time, spacing between slices, pixel matrix size and spacing size. The time interval (months) between scans was also recorded.

### Segmentation

**Figure 1:**
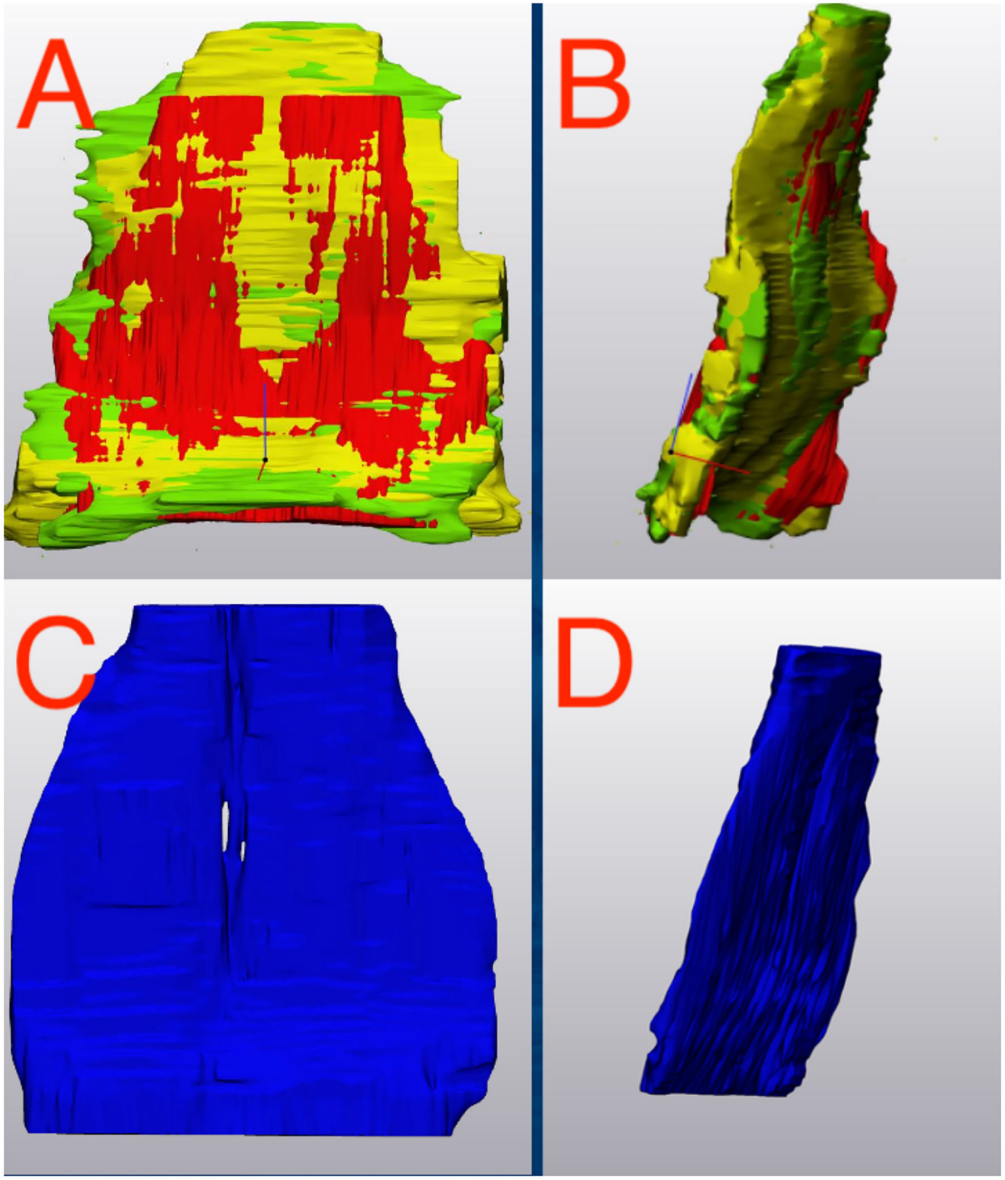
Deep Digital Flexor Tendon Segmentation. **(A,B)** The three segmentations (axial, sagittal and dorsal) are imported into a 3D manipulation program. **(A)** Dorsal view. **(B)** Sagittal view. A Boolean intersection is performed to select only the common components of all three views before a wrapping and smoothing algorithm is applied. **(C,D)** The final representative tendon used for analysis. **(C)** Dorsal view. **(D)** Sagittal view. Top of the image represents the proximal aspect of the tendon.

A single operator (HH) performed all the segmentations and was blinded to the MRI reports. DICOM files were imported into segmentation software (Mimics Ver 23.0, Materialize, Plymouth, MI). All 1.5 and 0.27 T images were segmented using the same methodology. 3D segmentation of the DDFTs for all horses at all time points was performed using a “livewire” segmentation algorithm using the PDW or the T1W 3D sagittal, axial and dorsal plane images. The livewire segmentation is a function of the Mimics segmentation software. The outline of the tendon is traced using a magnetic lasso which attracts to the interface between hypo and hyperintense signal regions. This is completed on each slice and the computer interpolates the 3D structure of the tendon from this data. Thus, each horse has their DDFT segmented in triplicate (once for each segmental plane). Segmentation was cropped to (1) the palmaroproximal aspect of the middle phalanx and (2) tangent to the most distal aspect of the distal interphalangeal joint, to maximize consistency in segmentation size and enhance repeatability. Only hypointense signal on the MRI scans was segmented; hyperintense lesions were excluded from our final model. Once segmented, volumetric tendon surface tessellation language (STL) files were created. These output files generate a 3D volume that can be rotated and manipulated to provide a 360-degree 3D view of the segmented tendon. These STL files were exported to 3D modeling software (3-Matic Ver 15.0, Materialize, Plymouth, MI). Thus, for each time point and tendon, 3 STL files were imported into the modeling software. In this software, the STL files maintain their original spatial relationship and therefore, a boolean intersection of all the three STL files was used to create a single, common, representative portion of the DDFT for the final analysis (Figure 1). These final tendon shapes are referred to as “parts” in the modeling software. These tendon parts were refined through a wrapping and smoothing algorithm prior to final analysis. The tendons were wrapped using a gap closing distance of 0.5 mm with the size of the triangles on the newly created surface (smallest detail function) equal to 1 mm. The parts were then smoothed using a smoothing factor of 0.7.

### Tendon Analysis

**Figure 2:**
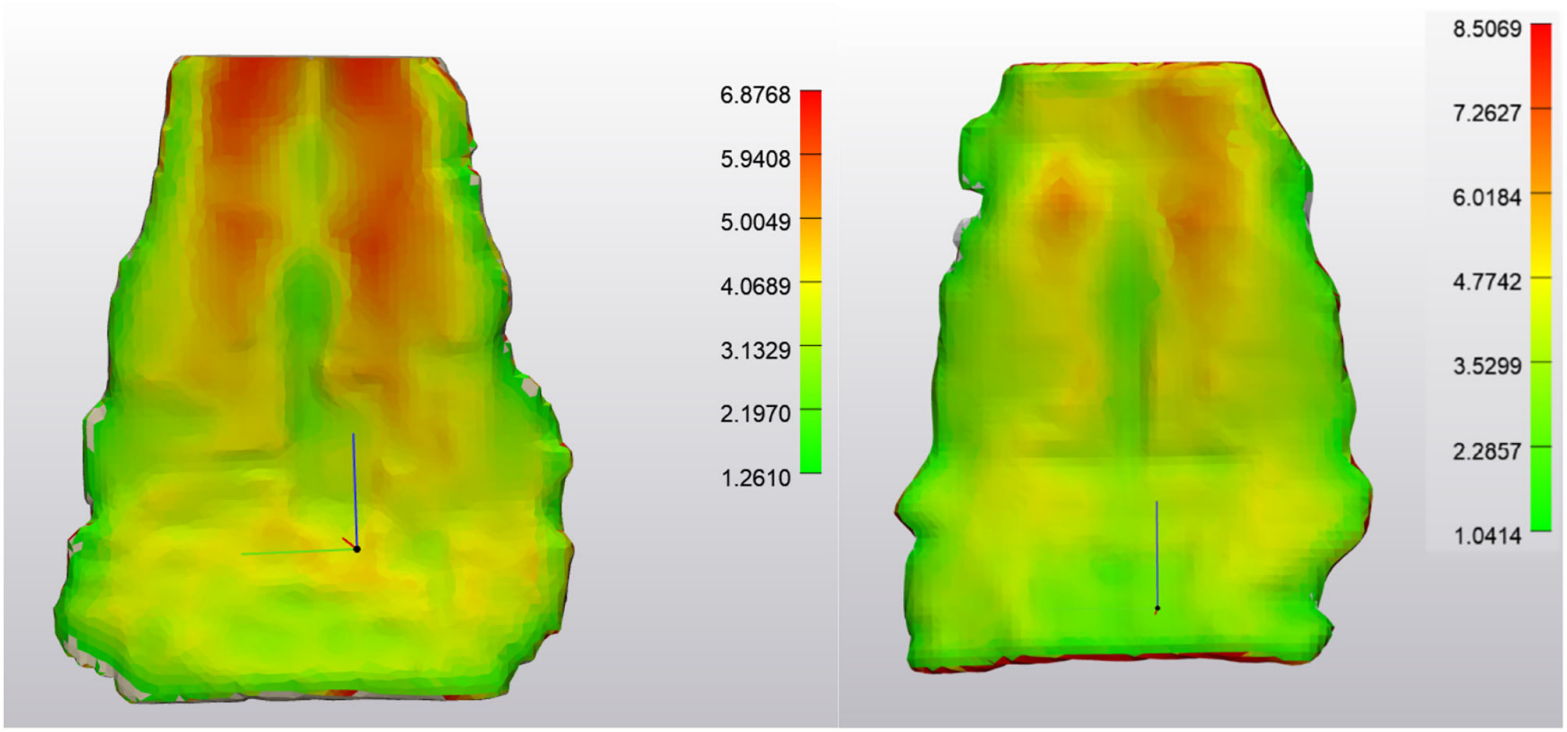
NORMAL Deep Digital Flexor Tendon. **(Left)** Dorsopalmar tendon thickness evaluation at first time point (after wrapping and smoothing algorithm applied). Measurements are in mm. Note the change in thickness between the bilobed tendon structure proximally to the flat distal portion of the segment. Thickness range is 1.26–6.88 mm. **(Right)** Tendon thickness evaluation from the same horse 7 months later. Thickness range 1.04–8.50 mm. Top of the image represents the proximal aspect of the tendon.

**Figure 3:**
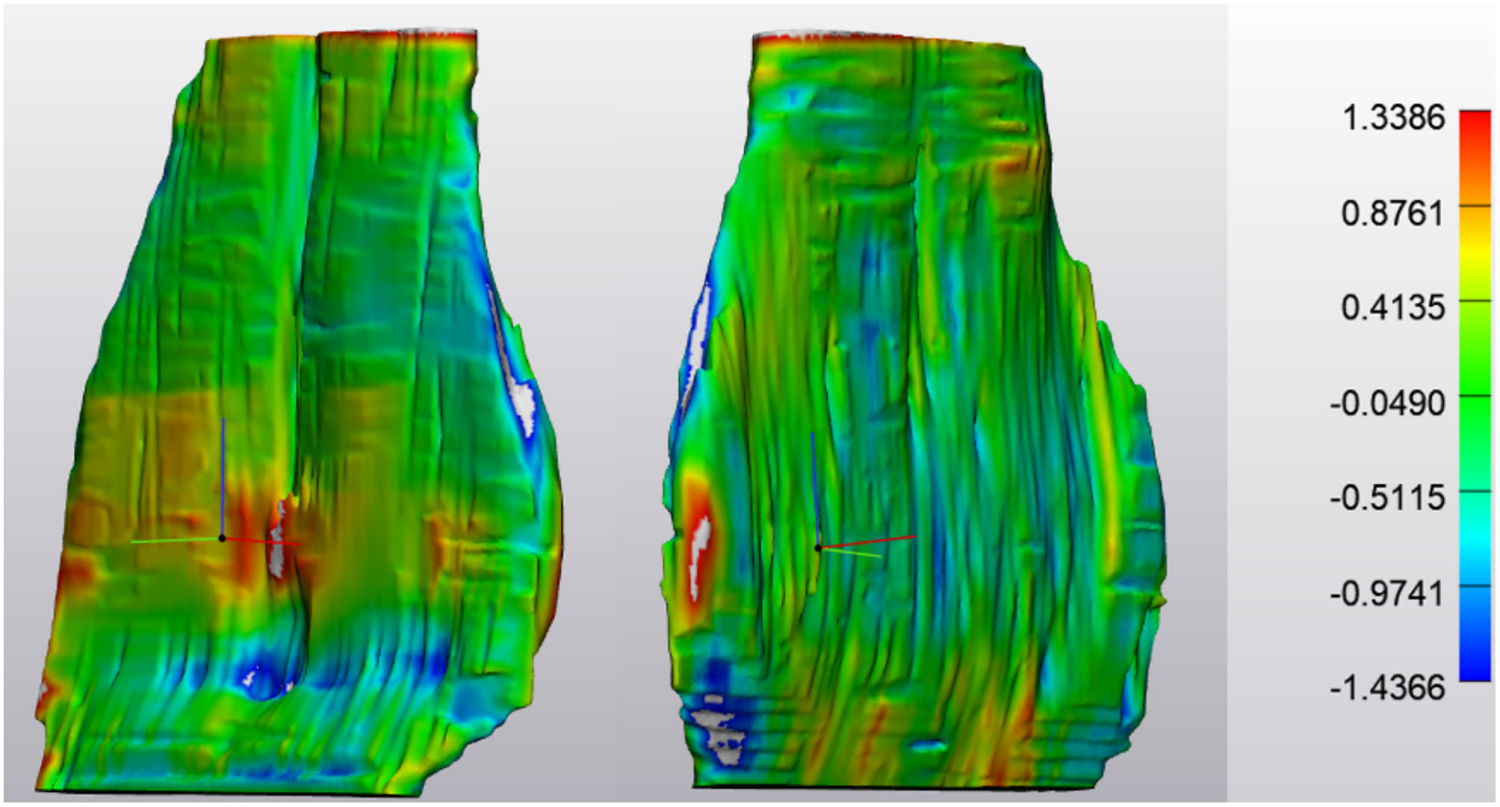
NORMAL Deep Digital Flexor Tendon. Analysis of the change in tendon size in one horse over 7 months (prior to application of wrapping and smoothing algorithms). Size change is limited to +1.3386 or −1.4366 mm. No significant pattern is observed. **(Left)** Obliqued dorsal view. **(Right)** Obliqued palmar view. Top of the image represents the proximal aspect of the tendon.

Outcome measures for each tendon included the tendon volume (mm^3^) and tendon thickness (mm, Figure 2). Tendon thickness varies throughout the tendon, therefore, minimum, median and maximum thickness values for each tendon were recorded. Based on each MRI scan, we acquired data for unaffected tendons (normal) and tendons with noted pathology (abnormal). In order to compare tendons at different time points, tendons were aligned using the global registration function using a distance threshold of 5.0 mm with 100 iterations and a 90% subsample threshold. The global registration is an algorithm embedded in the tendon analysis program (3-Matic). Once aligned, a “part comparison” was made between the two tendons (Figure 3). The part comparison is a point-based comparison between two datasets which has also been termed the Hausdorff Distance (18). This generates a 3D color map which visually identifies changes in the tendon thickness. This data can be downloaded into excel format, from which we can determine the minimum, median and maximum differences (mm) between the two parts. Negative values correspond to a reduction in thickness at any given location.

### Data Analysis

Data were evaluated for normality using the Shapiro Wilk test. Where normal, data are reported as mean ± standard deviation, when data are parametric, they are reported as median (range). Comparisons between non-parametric data were performed using the Wilcoxon rank sum test. Pearson correlation coefficients were calculated for comparison of MRI reports to computer analysis of thickness and volume. Odds ratios for changes in tendon thickness over time were calculated along with 95% confidence intervals. *P* < 0.05 was considered significant. Calculations were performed with MedCalc (MedCalc Ver 20.0, MedCalc Software Limited, Ostend, Belgium).

## Results

MRI examinations were acquired from 6 Warmblood horses. Sexes comprised of five geldings and one mare with a median age of 11.0 years (range 8–14 years). One horse had three scans using a high field system, 3 horses had 2 scans using a high field system, 1 horse had 2 scans using a low field system and 1 horse had a mixture of high and low field scans (Table 1). Five left limbs and 5 right limbs were imaged. All limbs imaged were forelimbs. Four horses had complete (left and right sets) of data for all scans. Two horses only had single limbs scanned at all time points (no contralateral comparison). In addition, 2 horses had 3 time points that were used in the analysis. One of these horses had a complete set of data from high field scans, the other had 2 low field scans followed by a high field (Table 1). The median time from onset of clinical signs to initial MRI scan was 3 weeks (range 1–3 months). The median time from first MRI scan to second MRI scan was 5 months (range 2–10 months). The time interval between the second and third MRI scans for the two horses who had three examinations was 3 and 6 months. For the horses who underwent high field (1.5 T) imaging using the PD sequences, the median slice thickness of acquired images was 3.2 mm (range 3 mm-5 mm), the repetition time was 3,072 (range 1,859–5,000), the echo time was 16.8 (range 10–45.6) and the space between slices was 3.6 mm (range 3–4.43 mm). For the low field (0.27 T) imaging using PDW and T1 W 3D sequences, the median slice thickness was 5.0 mm (range 2.97–5 mm), the repetition time was 1,000 (range 24–2002), the echo time was 24 (range 7–81) and the space between slices was 6 mm (range 2.97–6 mm). There was no significant difference in slice thickness (*p* = 0.36) between low and high field imaging or in the space between slices (*p* = 0.16). Images scanned using the low field system had a final resolution (pixel spacing size) of 0.7031 mm × 0.7031 mm with a pixel matrix of 256 × 256. Images scanned using the high field system had a 2.25× high final resolution (pixel spacing size) of 0.3125 mm × 0.3125 mm with a pixel matrix of 512 × 512.

**Table 1:** Summary data for each horse at each time point.

**Horse**	**Limb**	**Time**	**Median thickness (mm)**	**Volume (mm^3^)**	**Magnetic field strength (T)**	**MRI score**	**Clinical score**
1	LF	0	3.59	10,529.87	1.5		
		2 W	3.55	10,988.45	1.5	0	0
		3 M	3.15	9424.53	1.5	−1	−1
2	LF	0	2.09	5836.98	0.27		
		4 M	1.69	5992.24	0.27	1	0
		10 M	4.37	6308.82	1.5	0	−1
	RF	0	4.18	16,125.06	0.27		
		4 M	1.92	5616.88	0.27	−1	0
		10 M	4.24	16,107.65	1.5	−1	−1
3	LF	0	5.24	13,500.79	1.5		
		7 M	4.36	10,486.49	1.5	1	1
	RF^*^	0	4.82	16,549.50	1.5		
		7 M	4.34	16,164.58	1.5	0	0
4	LF	0 M	3.78	12,286.69	1.5		
		10 M	3.58	12,608.85	1.5	1	1
	RF	0 M	3.91	13,122.64	1.5		
		10 M	3.67	12,655.33	1.5	−1	1
5	LF	0 M	3.74	10,784.55	0.27		
		5 M	2.21	5953.20	0.27	0	0
	RF	0 M	2.32	6101.07	0.27		
		5 M	2.55	8487.66	0.27	0	0
6	RF	0 M	4.30	11,582.57	1.5		
		2 M	3.78	10,596.44	1.5	1	1

*Time points denote time since the original scan. MRI score and Clinical scores denote changes between time points; a recorded increase in severity of clinical signs or MRI pathology (−1), no change in clinical signs or similar MRI pathology (0) or an improvement in MRI pathology or clinical sign (+1).*

* *Denotes a normal DDFT that did not change between scans*.

**Figure 4:**
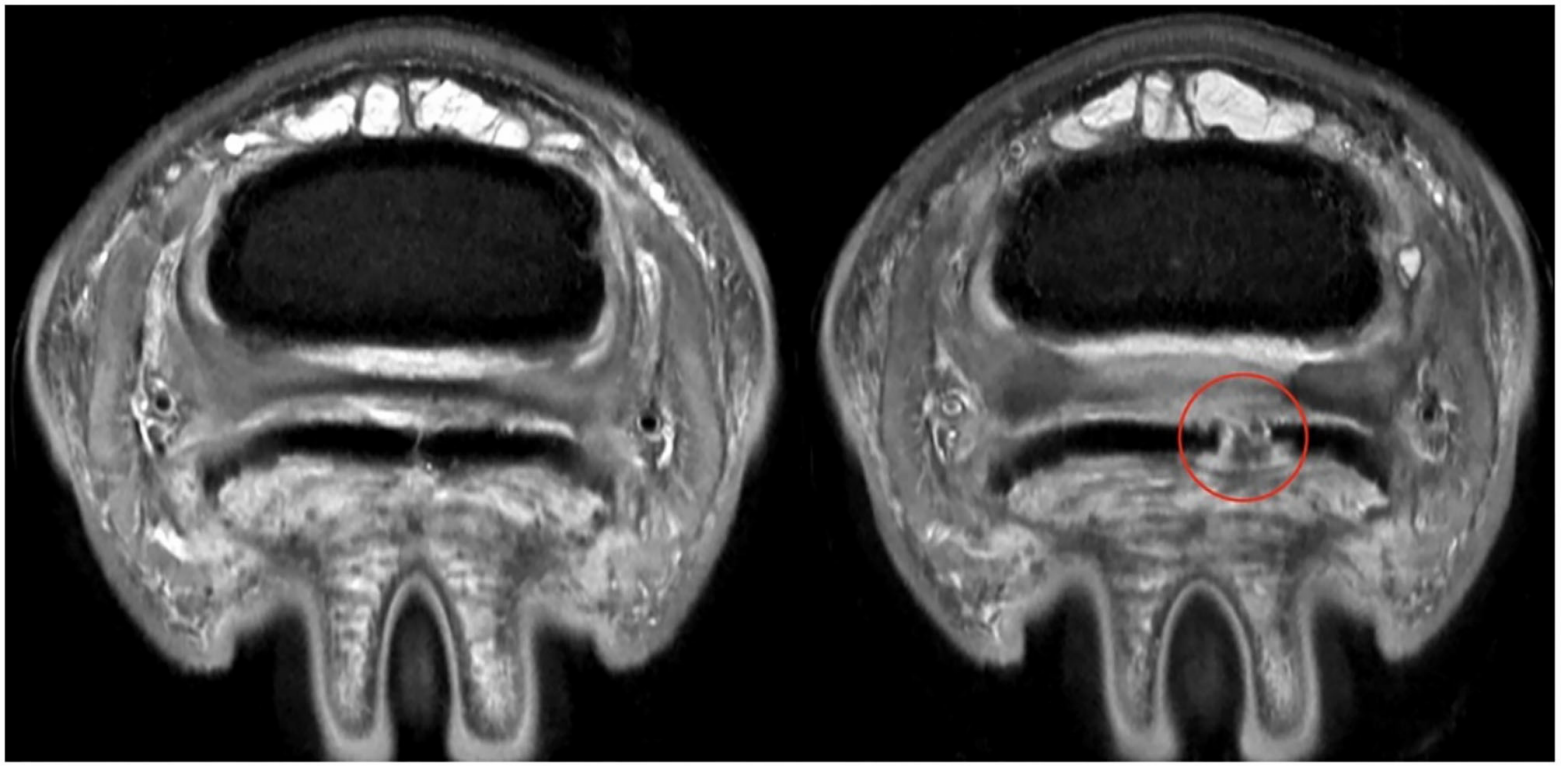
ABNORMAL Deep Digital Flexor Tendon. Axial section at same level of the foot for the same horse at two different time points. **(Left)** First time point, the delineation of the DDFT can be established. **(Right)** MRI scan 10 months later, demonstrating a complete and wide parasagittal split (red circle) in the lateral lobe of the DDFT. In this case, the lesion was NOT segmented with the tendon. Only intact portions of tendon were used for segmentation. This provides an absence of data in the DDFT for analysis purposes. The horse also experienced a worsening of clinical signs. Top of the image represents the dorsal aspect of the limb.

**Figure 5:**
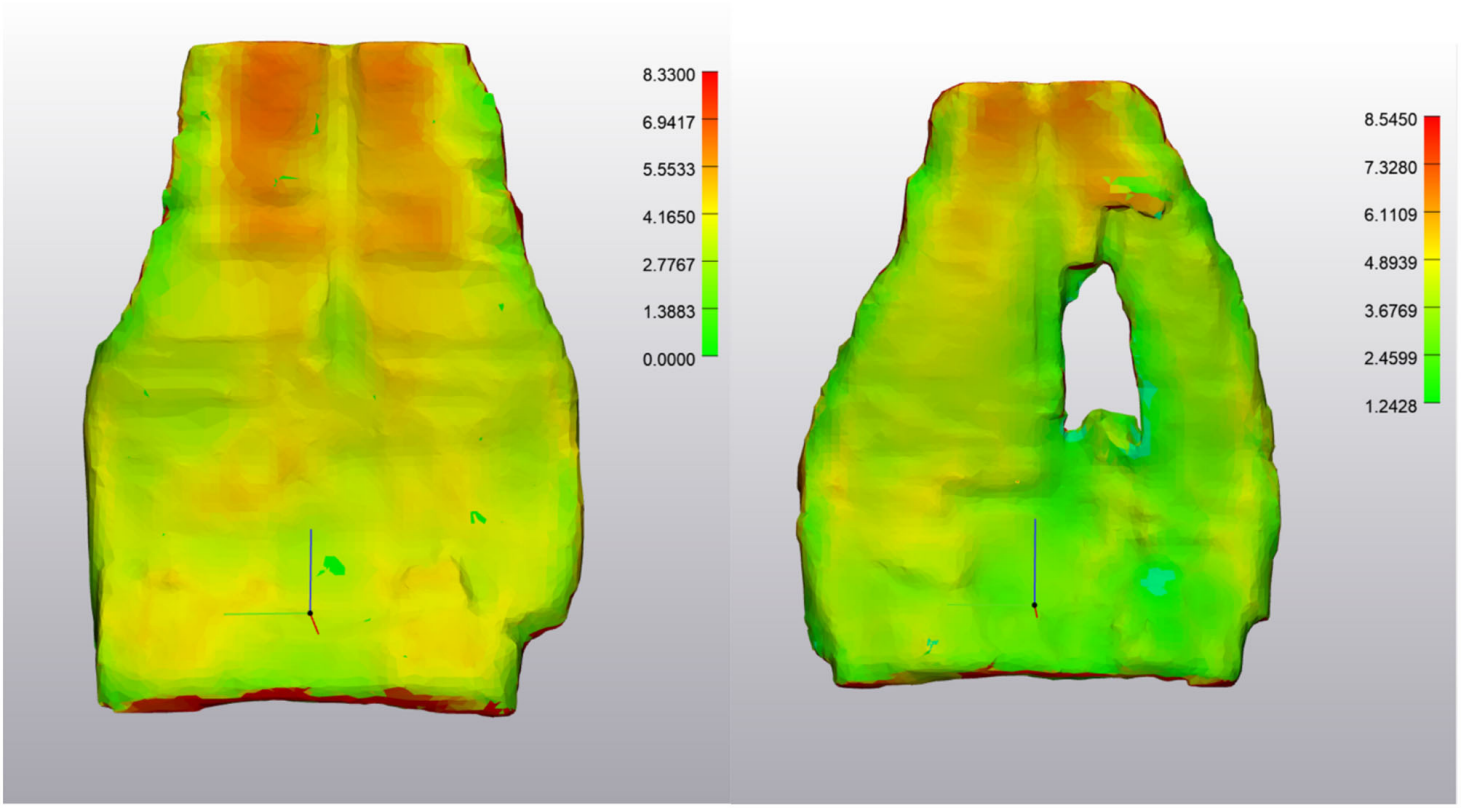
ABNORMAL Deep Digital Flexor Tendon. **(Left)** Tendon thickness evaluation at first time point. Measurements are in mm. Thickness range is 1.38–8.33 mm. **(Right)** Tendon thickness evaluation from the same horse 10 months later. Thickness range 1.24–8.54 mm, however, note the large absence of data associated with the tendon lesion and thinner normal tendon tissue surrounding the lesion. This is the same tendon as Figure 4. Top of the image represents the proximal aspect of the tendon.

**Figure 6:**
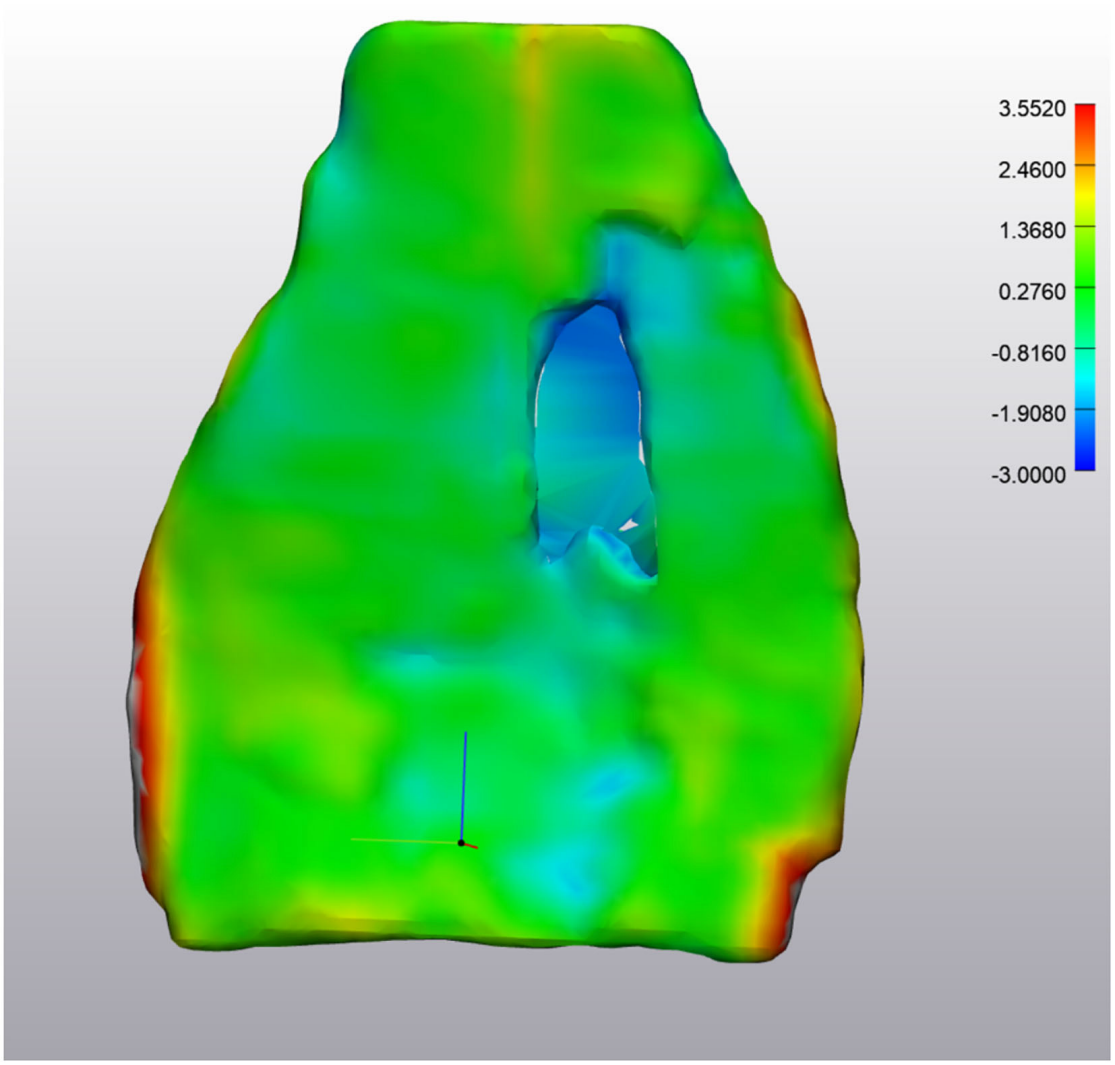
ABNORMAL Deep Digital Flexor Tendon. Analysis of the change in tendon size. The majority of the tendon appears green, indicating the change is minimal (−0.81 mm −1.36 mm difference). However, the large defect in the center is in excess of −2.0 mm difference. The red portions along the edges are segmentation artifacts due to minor inconsistencies in the segmentation model. Top of the image represents the proximal aspect of the tendon.

**Figure 7:**
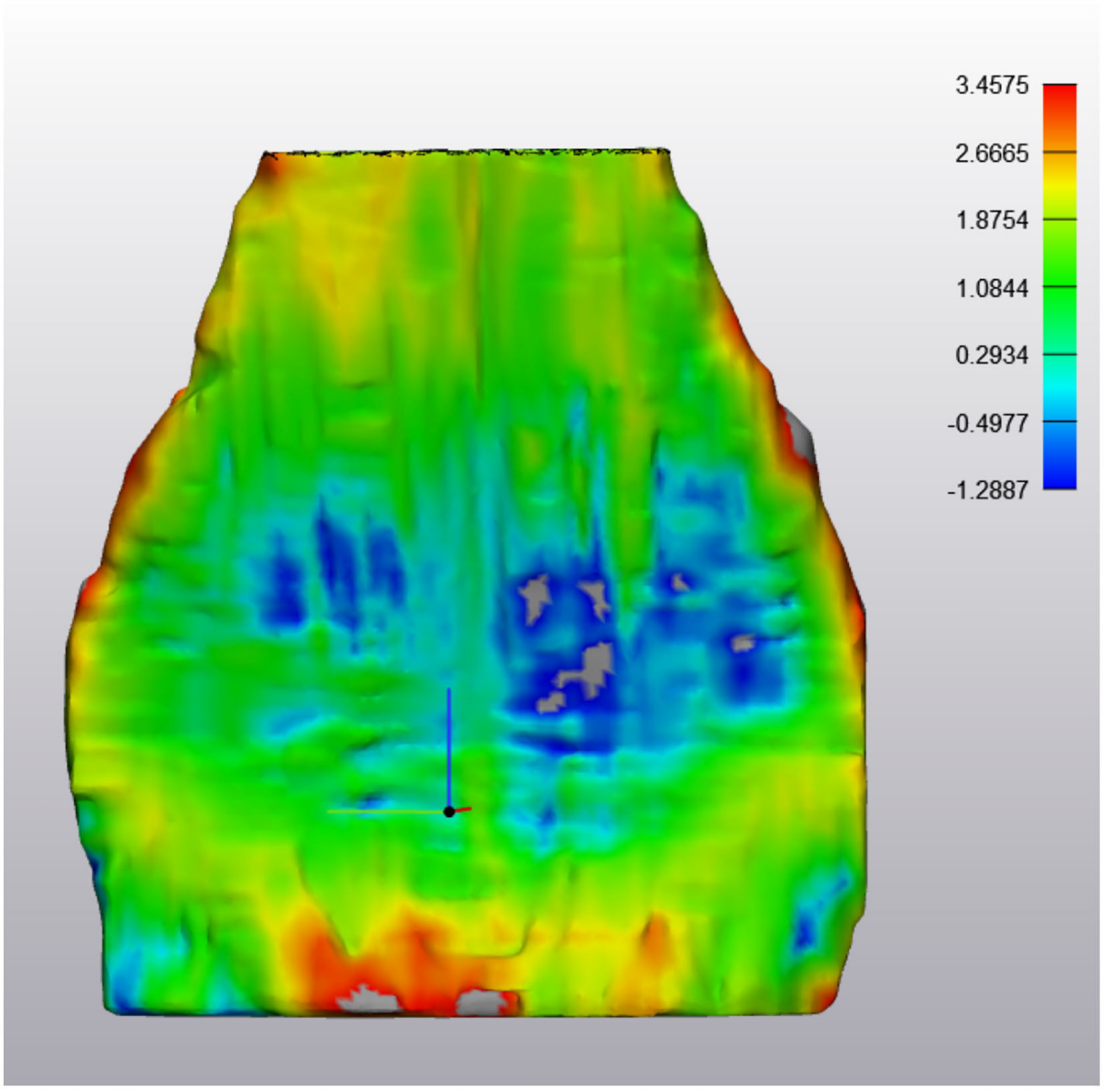
ABNORMAL Deep Digital Flexor Tendon. In this image, thinning has occurred in the mid region of the DDFT as it courses around the navicular bone over a 6 month period. However, both proximally (yellow) and distally (red) there is an increased in tendon thickness, indicating a dynamic volumetric response to tendon injury. Top of the image represents the proximal aspect of the tendon.

The lower end range limit for tendon thickness, identified distal to the navicular bone where the DDFT becomes thinner as it transitions to sheet-like varied between 0.16 and 1.74 mm. The upper end range limit for DDFT thickness, identified at the most proximal portion where the DDFT is bilobed, varied between 4.6 and 23.6 mm. Among abnormal tendons, differences of regional thickness ranged from −3.0 mm (indicating tendon thinning) to +14.3 mm (indicating tendon thickening, Figures 3–6). However, it should be noted, changes in DDFT size were non-uniform and demonstrated fluctuations throughout the tendon (Figure 7).

Only a single DDFT from one horse was classified as normal on two separate scans indicating no change in MRI appearance; all other DDFTs had abnormal pathological changes noted at all time points. This normal tendon had a part thickness between 1.55 and 8.98 mm, with a median value of 4.83 mm. The volume of the tendon was 16,550 mm^3^. At the second MRI scan 7 months later, the tendon had a thickness range of 1.03–8.46 mm with a median value of 4.35 mm and a volume of 16,164 mm^3^ (2.3% volume reduction). When comparing the thickness of the tendon between these 2 time points, the range of thickness change was between −1.44 to +1.34 mm with a median value of +0.36 mm.

For horses who had a DDFT with pathology (*n* = 21 tendons), the minimum tendon thickness was 0.76 ± 0.48 mm with a maximum value of 12.3 ± 6.3 mm. The median tendon thickness value was 3.5 ± 0.9 mm with a volume of 10,576 ± 3,698 mm^3^.

Three horses (4 DDFT's at various time points) had no changes in MRI and clinical scores between time points (recorded as 0), 3 horses (3 DDFTs at various time points) had concurrent improvements in MRI and clinical scores (recorded as +1), 2 horses (2 DDFTs at various time points) had concurrent deterioration in MRI score and clinical signs and 1 horse (2 DDFTs at various time points) had discordant MRI and clinical scores (Table 1). When comparing horses who had no change reported in MRI or clinical scores, the median tendon volume change was +2.6% (range −2.3 to +4.3%), the minimum change in part thickness between scans was −1.27 mm (range −0.79 to −1.43 mm) and the maximum change in thickness between scans was +2.55 mm (range 1.33 to +8.2 mm). The median value of the change in thickness between unchanged (score 0) tendons was 0.165 mm (range −0.07 to +0.36 mm).

For horses who experienced clinical improvement or an improvement in DDFT MRI outcomes, the median volume change was +8.5% (range +5.2 to +22.3%), the minimum change in part thickness between scans was −1.7 mm (range −1.0 mm to −2.2 mm), the maximum change in part thickness between scans was +2.0 mm (range +1.5 to +9.2 mm), and the median value of the change in thickness between improved tendons (score +1) was 0.8 mm (range −0.3 to +1.8 mm).

For horses who experienced a worsening of clinical signs or more significant MRI pathology (*n* = 3), the median volume change was +14.2% (range −3.6 to +287%), the minimum change in part thickness between scans was −1.3 mm (range −1.2 to −1.6 mm), the maximum change in part thickness between scans was +3.0 mm (range +2.1 to +14.3 mm), and the median value of the change in thickness between tendons with more severe pathology or clinical signs (score −1) was 0.21 mm (range +0.2 to +2.8 mm).

There was a positive correlation between MRI reports (improved or worsened pathology) and computer analysis of median thickness (*r* = 0.5752, *p* = 0.01), however, there was no significant correlation with tendon volume (*r* = 0.4558, *p* = 0.057).

When comparing tendons within each horse over each scan, horses reported to have improved (+1) or worsened (−1) clinically with comparable MRI changes are 39.0 times (95% confidence interval 1.3 to 1,191) more likely to demonstrate changes in tendon thickness by at least ±1.5 mm compared to horses reported to be static or who demonstrate no changes in tendon pathology (0) (*p* = 0.038).

## Discussion

The study was successful in segmenting a standardized part of the DDFT in normal and diseased tendons and establishing the volumetric appearance and thickness of the DDFT as it courses in the foot. The change in shape of the DDFT from a bilobed appearance proximally to a sheet like appearance as the tendon courses distally was easily visualized. We encountered difficulties in accurate segmentation of the distal insertion of the DDFT as it inserts on the distal phalanx. This could potentially be due to mild obliquity of the MRI slices or due to the fan-like expansion which contains cartilage at the insertion of the tendon (2, 19). Furthermore, there were challenges with the low field images which had a poorer resolution compared to the high field images which has previously been demonstrated (20). This created issues when manually segmenting the soft tissue structures as images become pixelated (low resolution) when attempting to accurately trace the tendon outline, to a lesser degree this was also encountered in high field images. Due to inconsistencies in this segmentation, the decision was made to crop the distal portion of the DDFT at a point where a tangent drawn from the most distal aspect of the distal interphalangeal joint intersected the DDFT. This procedure was additionally performed at the level of the most proximal aspect of the eminences of the middle phalanx. The section that remained for evaluation coincides with the most common locations of lesions within the deep digital flexor tendon (1). Furthermore, some of the scans were acquired using isotropic sequences, and some were acquired using anisotropic imaging. A previous study has demonstrated that anisotropic voxels have a negligible effect on the 3D reconstruction of bone geometric models, and we do not believe they had an impact on our tendon models in the present study (10). We do not make any specific recommendations in regard to the optimal sequences to use, although logically sequences with high spatial resolution with narrow slice thickness and slice spacing, would be optimal for 3D reconstructions. Although the high field sequences used PD sequences for axial, sagittal and coronal imaging, a complete set using a single sequence was not available in the low field system, hence the need to use multiple sequences.

The authors acknowledge there will be some variation in the foot position during recumbency when placed inside the coil in the magnet, which is further amplified if the horse is standing. Therefore, these variables will have an impact on the final thickness/part comparison that was performed. The position of the DDFT varies considerably during stance, and is under tension when fully weight bearing (19, 21, 22). Increasing the hoof angle, which occurs when positioning the horse for an MRI examination at our hospital, results in relaxation of the tendon and increases the cross-sectional area of the deep digital flexor tendon (19, 21–24). However, this positioning is necessary to decrease the probability of magic angle artifact (24). Magic angle artifact is observed as increased intratendinous signal intensity when the angle between the deep digital flexor tendon and the constant magnetic field approach 55° (24). Magic angle artifact has also been described in feet examined with low-field MRI; however, it is observed as an asymmetric appearance of the two lobes of the deep digital flexor tendon (25). Segmentation could enable a more accurate evaluation of the DDFT that was potentially overestimated due to the magic angle artifact.

Magic angle causes an artifactual increase in signal intensity, which can be mistaken for pathological change. In the current study, we were careful to exclude portion of tendon that had a hyperintense signal on our scans so that only hypointense tendon was used to create our 3D parts. The software we used can provide image fusion, so scans which reduce or highlight artifacts or lesions can be included/excluded in the final 3D model or segmented as a separate entity based on the desire of the operator. We aimed to focus this study solely on hypointense tendon signal from the scans, if core lesions were encountered, if they had a rim of hypointense tendon signal, they were included in the final 3D model. If the hyperintense signal contacted a tendon surface, the hyperintense region was excluded. Thus, the segmentation methodology we used, which aimed to segment regions of “normal tendon only,” exclusive of pathology, may explain the changes we noted in difference in tendon volume. It remains debatable if this is the correct methodology for tendon segmentation, however the technique allows for flexibility to include/exclude any pathology based on signal intensities and scan sequences used depending on the area of research.

It was noted that DDFTs which demonstrated thinning in one region were often thicker in other regions, perhaps due to compensation for changes in tensile forces (Figure 7). It has been shown that histological changes which reflect abnormal stress can occur (26, 27). These changes could potentially create an increase in the size of the tendon. Previous studies evaluating cross sectional area found that an increase in tendon size was only associated with core lesions (2, 27). While lesion type was not evaluated in the current study, it is interesting that the median tendon thickness and volume for tendons with lesions was less than in tendons reported as normal. This is in contrast with a previous study which found that lesions were not associated with thinning of the tendon (2, 27). The clinical implications and relevance of these findings remains to be determined. Further studies including histological evaluation to determine correlation with imaging findings should be performed to determine the significance of these findings.

Previous studies have shown that heel elevation effects maximal strain within the DDFT (22, 23). As inclusion of shoeing details was outside of the scope of the current study, future studies to investigate how long-term utilization of therapeutic farriery may affect the thickness of the DDFT could be performed.

### Limitations

The major limitations for this study are related to inaccuracies particularly around the periphery of the tendons during segmentation. Due to the low image numbers acquired during scanning resulting in slice numbers in the 30's, 3D multiplanar reconstruction using this data was challenging. Due to this, segmentation was performed in triplicate using each plane to provide separate DDFT outlines. In the future, scans using specific 3D sequences with isotropic voxel size, smaller slice gaps and higher resolution images could be acquired, however, this would often require a prolonged scan time. Furthermore, the use of lower resolution low field images also compromised the fidelity of DDFT recreation and analysis. Currently, it is unknown if any differences between low and high field MRI are due to the resolution issues between the two systems, operator error or an inherent variability in segmenting complex structures such as the tendon and further research would be required. In addition, the angle of the foot and DDFT tension which occur during limb positioning or between general anesthesia and standing MR image acquisition may have had an impact on the data. In the future, such studies are recommended to be conducted using high field imaging only. Furthermore, although clinical and MRI reports were coded according to changes noted in the DDFT between scans, many other structures including the navicular were reported to have changed. These may have been more significant than the DDFT changes. As such, it ids challenging to from a direct link between DDFT score changes and clinical outcomes when so many other structures can contribute to the global picture.

### Conclusion

The data presented here demonstrates that the DDFT can be segmented and volumetric studies based on size and shape can be performed using an *in silico* approach. There was a high correlation between lesions described on MRI reports and tendon thickness.

## Data Availability Statement

The raw data supporting the conclusions of this article will be made available by the authors, without undue reservation.

## Ethics Statement

The animal study was reviewed and approved by the University of Florida Institutional Animal Care and Use Committee. Written informed consent was obtained from the owners for the participation of their animals in this study.

## Author Contributions

KT-M: analysis and interpretation of the data, drafting the article, revising the article for intellectual content, and final approval of the completed article. AB: conception, design, acquisition of data, analysis and interpretation of the data, drafting the article, revising the article for intellectual content, and final approval of the completed article. HH: segmentation of images, analysis of the data, and final approval of the completed article. NW: acquisition of data, analysis and interpretation of the data, revising the article for intellectual content, and final approval of the completed article.

## Funding

This study was funded by the University of Florida Surgical Translational and 3D Printing Research Lab.

## Conflict of Interest

NW is employed by Equine Diagnostic Imaging, Inc. The remaining authors declare that the research was conducted in the absence of any commercial or financial relationships that could be construed as a potential conflict of interest.

## Publisher's Note

All claims expressed in this article are solely those of the authors and do not necessarily represent those of their affiliated organizations, or those of the publisher, the editors and the reviewers. Any product that may be evaluated in this article, or claim that may be made by its manufacturer, is not guaranteed or endorsed by the publisher.
